# Effectiveness of Interventions for Internet, Smartphone, and Gaming Addictions: Umbrella Review and Meta–Meta-Analysis

**DOI:** 10.2196/81705

**Published:** 2026-03-05

**Authors:** Minggang Zhang, Tong Lan, Tao Song, Xiaochun Wang

**Affiliations:** 1 School of Psychology, Shanghai University of Sport, 399 ChangHai Road, Shanghai, 200438, P.R. China, Shanghai, 200438, China, 86 18101817330; 2 Psychological Counseling Center, Nanchang Institute of Technology, Nanchang, China

**Keywords:** internet addiction, smartphone addiction, gaming disorder, umbrella review, meta–meta-analysis, intervention effectiveness, PRISMA

## Abstract

**Background:**

Digital addiction, including internet, smartphone, and gaming addiction, has emerged as a significant global health concern. Although a wide range of interventions has been evaluated, the fragmented and siloed nature of existing meta-analyses limits a clear understanding of the comparative effectiveness of different interventions across addiction subtypes.

**Objective:**

This umbrella review and meta–meta-analysis aimed to estimate the overall effectiveness of interventions for digital addiction and examine differential effects according to addiction subtype, intervention modality, study design, and control condition.

**Methods:**

A systematic search of 5 electronic databases (PubMed, Web of Science, Scopus, APA PsycInfo, and the Cochrane Library) was conducted from inception to June 24, 2025. Eligible studies were systematic reviews with meta-analyses evaluating interventions for internet, smartphone, or gaming addiction. Random-effects models were applied to synthesize standardized mean differences (SMDs). Methodological quality and certainty of evidence were assessed using A Measurement Tool to Assess Systematic Reviews 2 and the Grading of Recommendations Assessment, Development, and Evaluation framework.

**Results:**

A total of 29 meta-analyses, comprising 52 effect sizes and 66,530 participants, were included (*I*^2^=95.13%). Overall, interventions demonstrated a large and statistically significant effect in reducing digital addiction symptoms (SMD=–1.44, 95% CI –1.67 to –1.21; *P*=.003). Subgroup analyses indicated that the largest effects were observed for internet addiction (SMD=–1.70, 95% CI –1.99 to –1.42), followed by gaming addiction (SMD=–0.82, 95% CI –1.09 to –0.56) and smartphone addiction (SMD=–0.80, 95% CI –1.39 to –0.21). Exercise-based interventions, particularly those integrated with psychological approaches, showed large effect sizes (SMD=–3.14, 95% CI –4.30 to –1.97); however, this finding was based on a very limited number of effect sizes and should be interpreted cautiously. In addition, randomized controlled trials yielded larger effects than mixed study designs, and no-intervention controls were associated with larger effect sizes than mixed control conditions. The certainty of evidence was generally low.

**Conclusions:**

Interventions for digital addiction are effective, although their magnitude of benefit varies by addiction subtype and intervention modality. These findings support the use of tailored and multimodal intervention strategies while highlighting the need for more rigorous, high-quality, and balanced evidence across different forms of digital addiction.

## Introduction

In the digital era, behavioral addictions associated with the excessive use of internet-based technologies have emerged as major public health concerns. A recent global meta-analysis synthesizing 504 prevalence studies involving more than 2 million individuals across 64 countries reported pooled prevalence rates of 14.2% for internet addiction, 26.99% for smartphone addiction, and 6.04% for gaming addiction, with pronounced increases observed during the COVID-19 pandemic [1]. Consistent with these findings, pandemic-restricted analyses have shown prevalence rates rising to 30.7% for smartphone addiction and 10.6% for internet addiction [2,3], underscoring a growing public health burden that spans age groups, sexes, and cultural contexts. The clinical relevance of this burden is increasingly reflected in international diagnostic frameworks [4]. In 2019, the World Health Organization incorporated gaming disorder into the *International Classification of Diseases, 11th Revision* (*ICD-11*), formally assigning it to the “Disorders due to addictive behaviors” section [5]. Although internet and smartphone addiction have not yet been formally recognized within the *ICD-11*, accumulating evidence links all 3 conditions to emotional dysregulation, sleep disturbances, impaired academic and occupational functioning, and reduced quality of life [6-9].

Over the past decade, a broad range of interventions has been evaluated to address digital addictions, including cognitive behavioral therapy (CBT), group counseling, exercise training, mindfulness-based approaches, and multicomponent programs. A number of meta-analyses have synthesized evidence on the effectiveness of these interventions in reducing symptoms of internet, gaming, and smartphone addiction. For example, a meta-analysis of 59 randomized trials reported large overall effects for interventions targeting internet addiction and identified combined CBT and exercise interventions as particularly effective [10]. Similarly, exercise-based interventions have demonstrated substantial reductions in smartphone dependence among children and adolescents, with optimal effects observed for moderate-intensity aerobic exercise performed approximately 3 times per week over a 12-week period [11]. In the context of gaming disorder, a synthesis of 38 studies involving 9524 participants reported a moderate but highly heterogeneous treatment effect [12].

Despite these encouraging findings, the evidence base for interventions targeting digital addictions remains fragmented. Most meta-analyses to date focus exclusively on a single type of addiction, such as internet use, smartphone dependence, or gaming disorder, while adopting diverse outcome measures, inconsistent risk-of-bias frameworks, and varying inclusion criteria [12-14]. This fragmentation limits the comparability of intervention effects across different behavioral addictions and hinders the development of unified, evidence-based clinical guidelines. Consequently, a higher-level synthesis is needed to systematically integrate and compare findings across multiple meta-analyses.

Umbrella reviews, which synthesize evidence from multiple systematic reviews and meta-analyses, provide a robust methodological framework for addressing these challenges [15]. Although recent umbrella reviews have made initial progress, important gaps remain. For instance, one umbrella review pooled 5 meta-analyses addressing digital addiction, encompassing internet, smartphone, and gaming addiction and general screen use. While cognitive behavioral and exercise-based interventions emerged as promising, the study did not differentiate effects by addiction subtype or conduct quantitative meta–meta-analysis comparisons, limiting conclusions regarding relative effectiveness [16]. Another umbrella review focused on internet and smartphone addiction and confirmed potential benefits of psychotherapeutic and exercise-based interventions; however, it similarly lacked quantitative comparisons across addiction categories or intervention modalities [17].

To address these limitations, this study conducted an umbrella review and meta–meta-analysis of interventions targeting internet, smartphone, and gaming addictions. Specifically, we aimed to (1) quantify the overall effectiveness of major intervention modalities on addiction subtypes and (2) compare effect sizes across internet, smartphone, and gaming addictions. By providing a comparative, high-level synthesis of the current intervention landscape, this study sought to inform clinicians, researchers, and policymakers working to mitigate the psychosocial and health consequences of digital-age behavioral addictions.

## Methods

### Protocol and Registration

This umbrella review was preregistered in PROSPERO (CRD420251084061). This review was conducted and reported in accordance with the PRISMA (Preferred Reporting Items for Systematic Reviews and Meta-Analyses) 2020 statement [18].

### Search Strategy

Five electronic databases (PubMed, Web of Science, Scopus, APA PsycInfo, and the Cochrane Library) were systematically searched from inception to June 24, 2025. The search strategy included a combination of 4 key blocks of terms related to internet addiction, intervention, and meta-analyses. Table S1 in Multimedia Appendix 1 provides our electronic search strategy.

### Eligibility Criteria

We included systematic reviews with a meta-analysis examining the effects of interventions on internet, smartphone, or gaming addiction. For addiction subtype classification, studies were categorized as targeting internet addiction, smartphone addiction, or gaming addiction based on the primary addiction construct explicitly defined by the authors, regardless of the device used (eg, gaming on a smartphone). No reclassification of primary studies was performed to avoid introducing additional subjectivity.

Specific inclusion criteria were also defined using the population, intervention, comparator, outcome, and study design framework as follows:

- Population—reviews were eligible if they included studies that enrolled individuals with addictive or excessive or problematic internet or smartphone use or gaming.
- Intervention—reviews were eligible if they included studies that investigated any type of psychological, behavioral, or physical activity intervention, among other types, that was implemented for individuals as an addiction intervention. Studies evaluating pharmacological interventions were not eligible for inclusion.
- Comparator—reviews were eligible if they included controlled studies in which the addiction intervention was compared with any type of control group.
- Outcomes—reviews were eligible if they included studies that reported the results of at least 1 meta-analysis of an intervention for internet, smartphone, or gaming addiction at a postintervention time point vs controls. The outcome measures needed to use valid and reliable rating scales suitable for internet, smartphone, or gaming addiction. When more than one measure was described by a study, the primary outcome was used. If the primary outcome was not specified, the data from the measure reported first were extracted. Meta-analyses without any effect size were excluded.
- Study design—reviews were eligible if they assessed quantitative randomized controlled trials (RCTs) only and if they assessed a mixed evidence base (RCTs with other designs, such as non-RCTs and quasi-experimental studies). Reviews were included if they were published in English in peer-reviewed journals. Conferences, theses, and commentaries were not included.

### Data Extraction

Study characteristics and outcome measures were extracted by MZ and TL. Data entry was independently checked by TS after the data were entered into a Microsoft Excel spreadsheet. The following data from two domains were extracted: (1) study characteristics (author, year of publication, country, mean age of participants, number of included studies, and sample size [percentage of participants by gender]) and (2) interventions (addiction types, intervention types, control group conditions, and study design). All the extracted data were combined with those from the original review.

### Evaluations of Methodological Quality and Quality of Evidence

The methodological quality of eligible systematic reviews with meta-analyses was evaluated using a critical appraisal tool: A Measurement Tool to Assess Systematic Reviews 2 (AMSTAR 2) [19]. AMSTAR 2 contains 16 checklist items rated as “yes” (1 point), “partial yes” (0.5 points), and “no” (0 points) and is used to identify the risk of bias for systematic reviews that involve randomized and/or nonrandomized intervention studies. Among the AMSTAR 2 items, 7 are considered “critical” and 9 are considered “noncritical.” Reviews were scored as follows: “critically low” (>1 critical weakness and 3 noncritical weaknesses), “low” (>1 critical weakness and <3 noncritical weaknesses), “moderate” (1 critical weakness and <3 noncritical weaknesses), or “high” (no critical weaknesses and <3 noncritical weaknesses).

The certainty of evidence was assessed using the Grading of Recommendations Assessment, Development, and Evaluation (GRADE) approach [20]. Five domains were considered: risk of bias, inconsistency, indirectness, imprecision, and publication bias. Each outcome of internet-related addiction was assigned a certainty rating of “high,” “moderate,” “low,” or “very low” based on these criteria. Two authors independently performed the AMSTAR 2 and GRADE evaluations, with the third author solving any discrepancies through consensus.

### Statistical Analysis

#### Calculation of Effect Sizes

A meta-analysis was performed in this meta-review through Stata/MP (version 18.0; StataCorp) to calculate individual study and pooled effect sizes. To synthesize the findings on the effectiveness of interventions for internet, smartphone, and gaming addictions, meta-analyses were performed by combining the effect sizes and 95% CIs reported in each review via a random-effects model. Standardized effect sizes (standardized mean differences; SMDs) with 95% CIs were used as the effect measure for the meta-analyses. For consistency across studies, all effect sizes were converted such that negative values reflected beneficial intervention effects. All statistical tests were 2 tailed, and statistical significance was set at a *P* value of less than .05.

#### Testing Homogeneity and Potential Publication Bias

The heterogeneity of effect sizes was tested using the Cochran *Q* test and *I*^2^ statistic, which indicates heterogeneity in percentages [21]. Significant heterogeneity was deemed present if the *P* value of the *Q* test was less than .10, and 0% indicated no heterogeneity, whereas 25%, 50%, and 75% indicated low, moderate, and high heterogeneity, respectively. To consider potential publication bias, a visual inspection of a funnel plot was conducted, and we also applied the nonparametric trim-and-fill method using a random-effects model. The method was implemented using a linear estimator with imputation on the right side of the funnel plot, reflecting a scenario in which studies with smaller or nonsignificant (less negative) effect sizes may be underrepresented.

#### Subgroup Analyses

For subgroup analyses, we extracted subgroup-specific effect sizes and corresponding data as reported in the included systematic reviews and pooled these results across reviews. As in the original review, the subgroup analyses were planned according to addiction type, intervention type, control group type, and study design. We used a random-effects analysis to estimate the effect size and compare subgroups.

## Results

### Search Results and Study Characteristics

**Figure 1.**
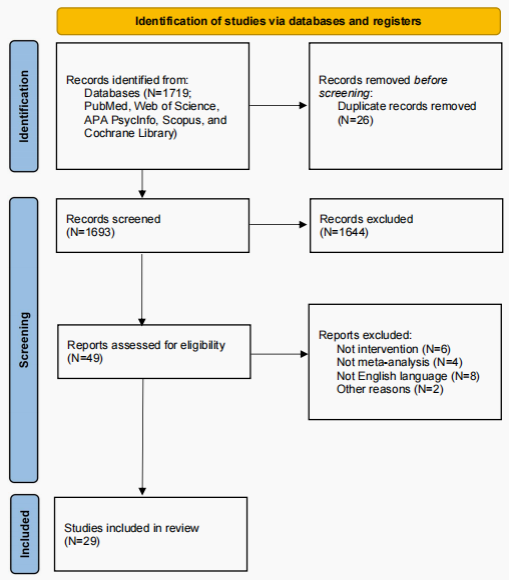
PRISMA (Preferred Reporting Items for Systematic Reviews and Meta-Analyses) flowchart.

A total of 1719 studies were identified through the database searches, and after the titles and abstracts were initially checked, 49 (2.9%) of the studies were included for full-text screening. We ultimately included 29 review and meta-analysis studies that met the full inclusion criteria. The PRISMA flowchart and checklist are presented in Figure 1 and Checklist 1, respectively, and the list of reasons for exclusion after full-text review is presented in Table S2 in Multimedia Appendix 1.

The eligible studies were published from 2017 to 2025 and were conducted in China (15/29, 51.7%), South Korea (6/29, 20.7%), Austria (2/29, 6.9%), Australia (1/29, 3.4%), Lithuania (1/29, 3.4%), Spain (1/29, 3.4%), the United States (1/29, 3.4%), Norway (1/29, 3.4%), and Turkey (1/29, 3.4%). Of these 29 studies, 18 (62.1%) assessed the intervention effect on internet addiction by calculating 38 effect sizes, 8 (27.6%) assessed the intervention effect on internet gaming addiction by calculating 10 effect sizes, and 4 (13.8%) assessed the intervention effect on smartphone addiction by calculating 4 effect sizes. A total of 31 effect sizes were calculated to assess psychological interventions for overall digital addiction, 14 effect sizes were calculated to assess exercise-based interventions, and 7 effect sizes were calculated to assess other types of interventions. In total, 41.4% (12/29) of the studies conducted a meta-analysis of RCTs, 58.6% (17/29) of the studies assessed mixed study designs, 24.1% (7/29) of the studies specifically assessed no-intervention controls, and 75.9% (22/29) of the studies assessed mixed controls. A summary of the participant demographics is provided in Table S3 in Multimedia Appendix 1.

Regarding the methodological quality and quality of evidence of all included studies, the percentages of studies with low and critically low methodological quality were 13.8% (4/29) and 86.2% (25/29), respectively, according to the AMSTAR 2 score (Table S4 in Multimedia Appendix 1). The percentages of reviews with moderate, low, and very low quality of evidence were 3.4% (1/29), 20.7% (6/29), and 75.9% (22/29), respectively, according to the GRADE score (Table S5 in Multimedia Appendix 1).

**Figure 2.**
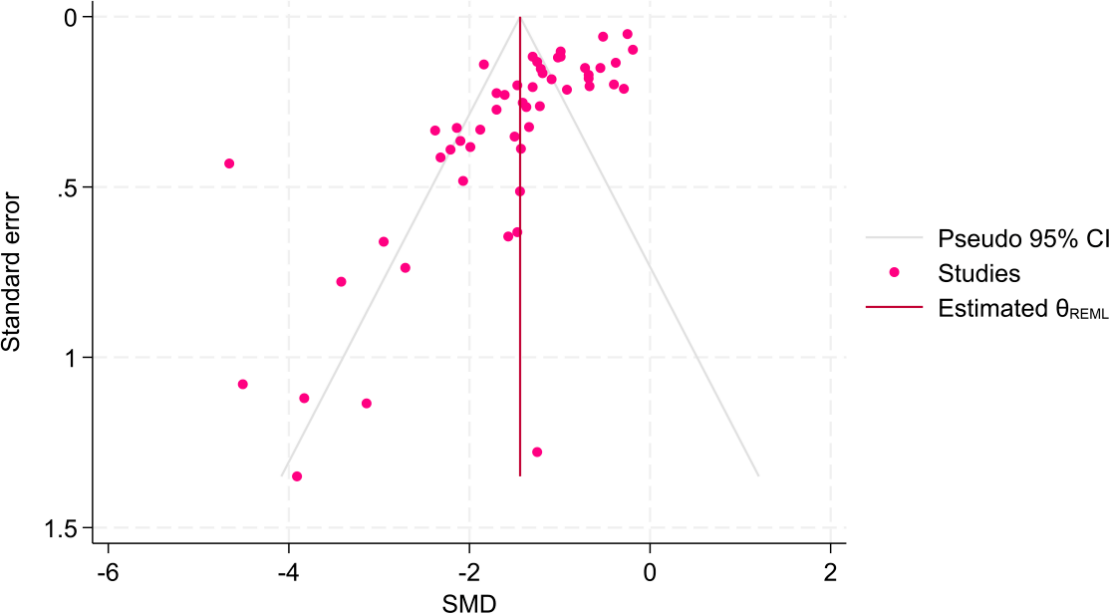
Funnel plot of all included effect sizes. REML: random effect model; SMD: standardized mean difference.

The visual and statistical results indicated significant asymmetry in the funnel plot (β=–3.00; *P*<.001), suggesting potential publication bias or small-study effects (Figure 2). The trim-and-fill procedure identified 14 potentially missing studies, resulting in a total of 66 studies (n=52, 78.8% observed; n=14, 21.2% imputed; Figure S1 in Multimedia Appendix 1). The observed pooled effect size was SMD=–1.439 (95% CI –1.67 to –1.21). After imputing the potentially missing studies, the adjusted pooled effect size was SMD=–1.09 (95% CI –1.38 to –0.81). Although the adjusted effect size was smaller in magnitude, it remained statistically significant, indicating a robust intervention effect despite potential publication bias.

### Meta–Meta-Analysis Results

**Figure 3.**
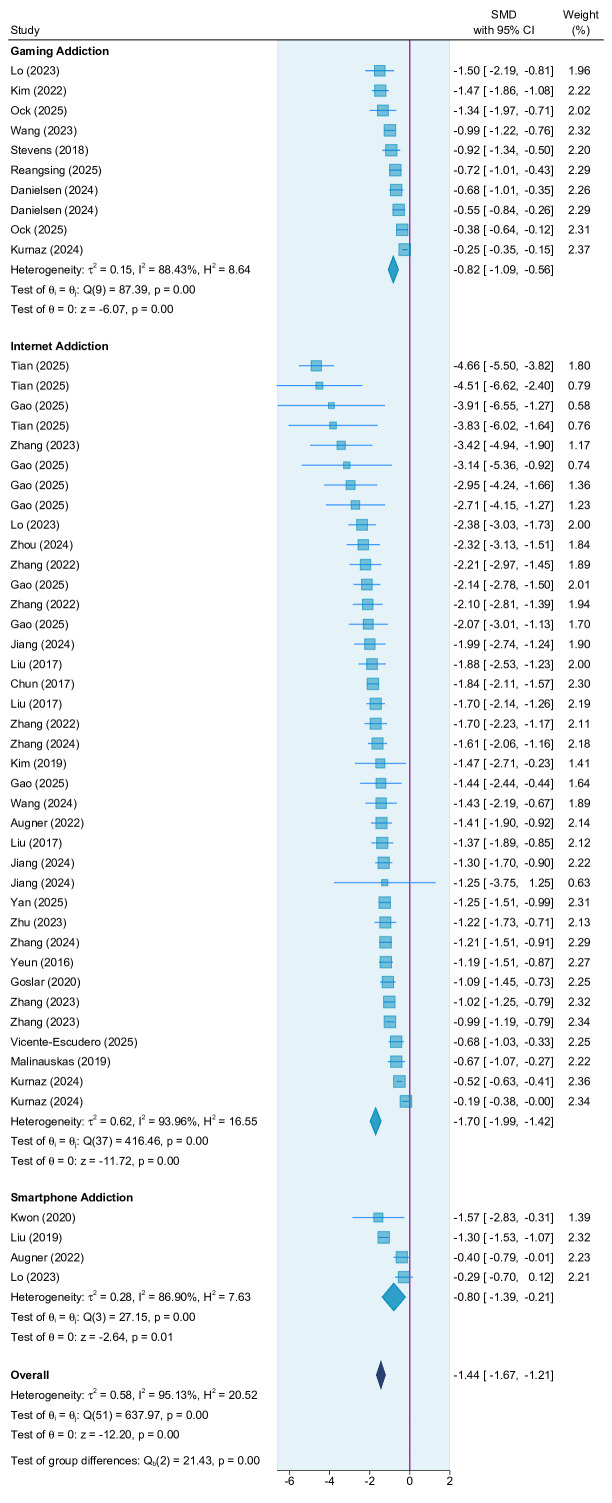
Forest plot for overall addiction and subaddictions [10,12,13,22-47]. SMD: standardized mean difference.

The overall analysis revealed that interventions significantly reduced digital addiction symptoms, with an SMD=–1.44 (95% CI –1.67 to –1.21; *P*=.003; *I*^2^=95.13%; Figure 3 [10,12,13,22-47]). Table 1 summarizes the results of the overall and subgroup meta-analyses. For different addiction types, the subgroup analysis revealed a significant effect (*Q*_b_=21.43; *df*=2; *P*=.003), and the interventions had the largest reducing effect on internet addiction (SMD=–1.70, 95% CI –1.99 to –1.42), followed by internet gaming addiction (SMD=–0.82, 95% CI –1.09 to –0.56) and smartphone addiction (SMD=–0.80, 95% CI –1.39 to –0.21).

**Table 1.** Results of overall and subgroup meta-analyses.

	Number of effects	SMD^a^ (95% CI)	*I*^2^ (%)	Test of subgroup differences	
				*Q*_b_ (*df*)	*P* value
Overall	52	–1.44 (–1.67 to –1.21)	95.13	—^b^	—
Subgroup analysis of all data					
Addiction				21.43 (2)	.003
Internet addiction	38	–1.7 (–1.99 to –1.42)	93.96		
Internet gaming addiction	10	–0.82 (–1.09 to –0.56)	88.43		
Smartphone addiction	4	–0.8 (–1.39 to –0.21)	86.9		
Intervention				58.68 (5)	.002
Psychological	31	–1.16 (–1.38 to –0.95)	92.53		
Exercise	14	–2.01 (–2.52 to –1.50)	93.9		
Exercise+psychological	2	–3.14 (–4.30 to –1.97)	—		
Biofeedback	2	–2.7 (–5.91 to 0.51)	88.6		
Behavioral	2	–0.46 (–0.65 to –0.26)	—		
Electrotherapy	1	–1.25 (–3.75 to 1.25)	—		
Study design				11.89 (1)	.008
RCT^c^	29	–1.83 (–2.20 to –1.46)	91.7		
Mixed	23	–1.06 (–1.29 to –0.82)	94.05		
Control				17.97 (1)	.007
No intervention	16	–2.45 (–3.04 to –1.86)	85.65		
Mixed	36	–1.12 (–1.30 to –0.93)	92.12		
Subgroup analysis for internet addiction					
Intervention				13.33 (4)	.01
Psychological	20	–1.34 (–1.63 to –1.06)	91.6		
Exercise	13	–2.08 (–2.63 to –1.53)	92.87		
Exercise+psychological	2	–3.14 (–4.30 to –1.97)	—		
Biofeedback	2	–2.7 (–5.91 to 0.51)	88.6		
Electrotherapy	1	–1.25 (–3.75 to 1.25)	—		
Control				12.44 (1)	.006
No intervention	16	–2.45 (–3.04 to –1.86)	85.65		
Mixed	22	–1.31 (–1.54 to –1.08)	90.41		
Study design				9.22 (1)	.005
RCT	24	–2.05 (–2.45 to –1.65)	88.14		
Mixed	14	–1.24 (–1.57 to –0.92)	94.13		
Subgroup analysis for gaming addiction					
Intervention				6.43 (1)	.01
Psychological	8	–0.93 (–1.24 to –0.62)	88.37		
Behavioral	2	–0.46 (–0.65 to –0.26)	—		
Study design				0.36 (1)	.55
RCT	3	–1.02 (–1.75 to –0.28)	83.03		
Mixed	7	–0.77 (–1.06 to –0.49)	88.64		
Subgroup analysis for smartphone addiction					
Intervention				23.60 (1)	.005
Psychological	3	–0.41 (–0.68 to –0.13)	—		
Behavioral	1	–1.3 (–1.53 to –1.07)	—		
Study design				0.00 (1)	.99
RCT	2	–0.81 (–1.80 to 0.18)	94.26		
Mixed	2	–0.82 (–1.93 to 0.28)	66.68		

a SMD: standardized mean difference.

b Not applicable.

c RCT: randomized controlled trial.

For the intervention types, the overall subgroup analysis revealed significant differences in efficacy among the included interventions (*Q*_b_(5)=58.68; *P*=.002). Although only 2 effect sizes were calculated to assess the efficacy of a psychological intervention plus exercise, this intervention had the greatest effect on overall addiction (SMD=–3.14, 95% CI –4.30 to –1.97), followed by biofeedback (SMD=–2.70, 95% CI –5.91 to 0.51) and exercise (SMD=–2.01, 95% CI –2.52 to –1.50) interventions. Specifically, the interventions also had significant effects on reducing addictive symptoms for internet addiction (*Q*_b_(4)=13.33; *P*=.01), internet gaming addiction (*Q*_b_(1)=6.43; *P*=.01), and smartphone addiction (*Q*_b_(1)=23.60; *P*=.005). For internet addiction, the intervention with the largest reducing effect was psychological plus exercise (SMD=–3.14, 95% CI –4.30 to –1.97), followed by biofeedback (SMD=–2.70, 95% CI –5.91 to 0.51) and exercise (SMD=–2.08, 95% CI –2.63 to –1.53) interventions. For internet gaming addiction, only 2 types of interventions were assessed. Compared with those of the behavioral intervention, the larger effect sizes of the psychological intervention indicated that it had greater effects on reducing addictive symptoms (SMD=–0.93, 95% CI –1.24 to –0.62). Finally, for smartphone addiction, only 3.4% (1/29) of the studies assessed a behavioral intervention and reported greater effects than those of a psychological intervention (SMD=–0.41, 95% CI –0.68 to –0.31).

With respect to study design, the subgroup analysis revealed that RCTs found a significantly greater effect in reducing overall addictive symptoms (SMD=–1.83, 95% CI –2.20 to –1.46) and internet addiction symptoms (SMD=–2.05, 95% CI –2.25 to –1.65) but not smartphone addiction (*Q*_b_(1)=0.00; *P*=.99) or internet gaming addiction (*Q*_b_(1)=0.36; *P*=.55) symptoms.

For the control conditions, the subgroup analysis revealed that a no-intervention control had a significant and greater effect on reducing overall and internet addiction with the same included effect sizes (SMD=–2.45, 95% CI –3.04 to –1.86) than a mixed control. No subgroup analyses were conducted for internet gaming addiction or smartphone addiction, as only 1 control was included.

The results of the subgroup analyses for different addiction types, intervention types, study designs, and control group types are presented as forest plots in Figures S2 to S11 in Multimedia Appendix 1.

## Discussion

### Principal Findings

In this umbrella review and meta–meta-analysis, evidence from 29 systematic reviews and meta-analyses was synthesized to evaluate the effectiveness of interventions targeting internet, smartphone, and gaming addictions. Overall, interventions demonstrated large and statistically significant effects in reducing digital addiction symptoms. Among the 3 addiction subtypes, interventions targeting internet addiction yielded the largest pooled effect, whereas those targeting gaming and smartphone addiction produced more modest but still clinically meaningful reductions. These findings support the overall efficacy of interventions for digital behavioral addictions and suggest that responsiveness to intervention may differ across addiction subtypes. Notably, to our knowledge, this study represents the first quantitative umbrella synthesis that directly compares effect sizes across different categories of digital addiction, thereby providing a more granular and comparative perspective on intervention effectiveness.

Our findings indicate clear differences in intervention effects across addiction subtypes. Interventions for internet addiction showed the largest pooled effect (SMD=–1.70), followed by interventions for gaming addiction (SMD=–0.82) and smartphone addiction (SMD=–0.80). These results are broadly consistent with those of prior meta-analyses reporting moderate effects for gaming disorder and smartphone addiction [11,12], while extending previous work by demonstrating the comparatively greater responsiveness of internet addiction to intervention. One potential explanation is that internet addiction, often conceptualized as a broader and more heterogeneous construct, encompasses diverse behaviors such as social media use, web browsing, and streaming, which may be more amenable to general behavioral regulation strategies [48,49]. In contrast, gaming and smartphone addiction may involve more entrenched reward-based and habit-forming mechanisms frequently embedded within immersive or socially reinforced contexts, thereby posing greater challenges for behavior change [50,51]. These distinctions underscore the importance of tailoring intervention strategies to the specific behavioral and cognitive characteristics of each addiction subtype.

With respect to intervention modalities, our results highlight a notable and often underemphasized finding. Exercise-based interventions, both as stand-alone approaches and in combination with psychological therapies, were associated with particularly large effect sizes. The combined psychological and exercise intervention demonstrated the largest pooled effect (SMD=–3.14), followed by exercise-only interventions (SMD=–2.01) and biofeedback-based approaches (SMD=–2.70). This pattern diverges from much of the existing literature, which has predominantly emphasized cognitive behavioral and psychotherapeutic interventions [17]. Nevertheless, our findings are consistent with emerging evidence supporting the benefits of physical activity for reducing internet and smartphone addiction symptoms [14,22]. Potential mechanisms underlying these effects include improvements in executive functioning, stress regulation, emotional control, and sleep quality [52,53]. At the same time, the strong performance of psychological-only interventions reinforces their established role in treatment and suggests that psychological and exercise-based modalities may exert complementary effects.

Subgroup analyses further revealed substantial heterogeneity in intervention effects not only across addiction subtypes but also within each subtype, depending on intervention modality, study design, and control condition. For internet addiction, combined psychological and exercise-based interventions yielded the largest effects. In contrast, behavioral interventions appeared more effective than psychological interventions for smartphone addiction, aligning with prior evidence regarding the role of physical activity in enhancing self-regulation and reducing screen-based behaviors [14,22]. For gaming addiction, relatively larger effects were observed for psychological interventions compared with behavioral approaches. These patterns support the increasingly accepted view that different forms of digital addiction may be underpinned by distinct neurocognitive and behavioral profiles and, therefore, require intervention strategies tailored to the specific structure and reinforcement mechanisms of each disorder [51].

Beyond intervention modality, methodological characteristics, including study design and control condition, emerged as important moderators of treatment effects. Across all 3 addiction subtypes, RCTs yielded larger effect sizes than non-RCT or mixed designs, with this difference being most pronounced for internet addiction. This finding reinforces the importance of experimental rigor in evaluating intervention efficacy [15,54]. Regarding control conditions, studies using no-intervention or waitlist controls consistently reported larger effect sizes than those using active or mixed control groups, particularly for internet and smartphone addiction. While this pattern may partly reflect expectancy effects or the absence of placebo balancing, it also raises concerns about potential effect size inflation in less rigorously controlled designs [55,56]. Similar trends have been documented in psychotherapy research, where studies using no-treatment controls often report larger effects than those using active or placebo comparators [57]. These observations highlight the necessity of incorporating well-designed active or attention-matched control conditions in future trials to enhance interpretability and comparability.

Although the meta–meta-analytic findings provide strong support for the effectiveness of interventions, caution is warranted when interpreting the apparent superiority of certain intervention types. In this synthesis, interventions for internet addiction accounted for a disproportionately large share of the available effect sizes, whereas smartphone addiction and gaming addiction were represented by substantially fewer meta-analyses and primary studies. This imbalance likely reflects historical research priorities and differences in diagnostic recognition, particularly given the relatively recent inclusion of gaming disorder in the *ICD-11* [5,58]. However, such disparities limit the robustness of cross-subtype comparisons. Specifically, the smaller evidence base for smartphone and gaming addiction may reduce statistical power, increase sensitivity to individual study characteristics, and obscure true intervention effects. Consequently, the comparatively lower pooled effects observed for these subtypes should not be interpreted as definitive evidence of inferior treatment efficacy. Addressing this limitation will require greater investment in high-quality, subtype-specific intervention research.

Several contextual factors should also be considered when interpreting these findings. First, most included meta-analyses were conducted in middle- and high-income countries, which may limit the generalizability of the results to settings affected by the digital divide, where access to digital technologies and intervention resources differs substantially. Second, reverse causation cannot be fully excluded, as individuals with more severe addiction symptoms may be more likely to participate in intervention studies, potentially inflating observed effects, particularly in non-RCT designs. Moreover, intervention effectiveness may vary by gender, age, and regional context; however, most included reviews did not report stratified analyses for these variables. Future studies should address these gaps to improve the external validity of digital addiction interventions.

Conceptual overlap among digital addiction constructs should also be considered. In practice, behaviors such as gaming may occur on multiple platforms, including smartphones and computers, which may blur the boundaries among internet, smartphone, and gaming addiction. This overlap could partially attenuate observed differences in intervention effectiveness across addiction subtypes and should be taken into account when interpreting comparative results. Future research should also establish a more detailed distinction among these potential impacts. Finally, the substantial heterogeneity observed in the overall analysis (*I*^2^=95.13%) likely reflects multiple sources of variability beyond intervention type. These include differences in diagnostic criteria and measurement instruments, variation in symptom severity and age composition of samples, and cultural and regional differences in digital use patterns. Additionally, the inclusion of both RCTs and mixed study designs may have further contributed to heterogeneity. Such variability underscores the need for cautious interpretation of pooled estimates.

This study has several key strengths. First, it is the first umbrella review and meta–meta-analysis to systematically compare the effectiveness of interventions across 3 distinct digital addiction types, addressing a major gap in prior literature. Second, it evaluated multiple intervention modalities, offering a broader understanding of the treatment landscape beyond the dominant CBT-centric models. Third, by applying rigorous tools such as AMSTAR 2 and GRADE, it appraised not only effect sizes but also the credibility of the evidence. Notably, the use of these tools revealed that all of the included meta-analyses were rated as low or critically low in methodological quality, and most effect estimates were graded as low or very low in certainty. This outcome reflects the stringent criteria of both appraisal systems. AMSTAR 2 places strong emphasis on protocol registration, risk-of-bias assessment in primary studies, and reporting transparency—criteria often underreported in existing meta-analyses [19]. Similarly, GRADE tends to downgrade evidence derived from observational studies or indirect comparisons and penalizes for inconsistency, imprecision, and publication bias, which were common across the included reviews. While these findings do not negate the observed intervention effects, they underscore the need to improve the methodological transparency and consistency of future meta-analyses in this domain [59].

Nevertheless, several limitations must be acknowledged. All of the included meta-analyses were rated as low or critically low in methodological quality, and the overall certainty of evidence was predominantly classified as very low. Substantial heterogeneity and indications of publication bias further limit the interpretability and generalizability of the findings. In addition, studies focusing on gaming and smartphone addiction were underrepresented relative to those addressing internet addiction, and several intervention approaches were supported by only a small number of studies. These limitations highlight the need for more rigorous, targeted, and subtype-specific intervention research. Finally, the larger effect sizes observed in studies using no-intervention controls are consistent with prior psychotherapy research and likely reflect expectancy and placebo-related effects. Future intervention trials should prioritize the use of active or attention-matched control conditions to reduce bias and improve the interpretability of treatment effects.

Despite these limitations, the findings of this study have important implications for clinical and applied practice. Given the comparatively strong effects observed for integrated interventions, particularly those combining psychological therapy with exercise, clinicians and educators may consider adopting multimodal treatment strategies, especially in school-based or youth-oriented settings. Moreover, the observed benefits of stand-alone exercise and biofeedback interventions underscore the potential value of nonpharmacological, resource-efficient approaches that may be scalable in low-resource or community contexts. Tailoring interventions to specific addiction subtypes, developmental stages, and cultural backgrounds while maintaining rigorous study designs and standardized outcome measures may further enhance the effectiveness and ecological validity of future digital addiction interventions.

### Conclusions

This umbrella review demonstrates that interventions targeting internet, smartphone, and gaming addictions are generally effective. Integrated psychological-exercise interventions appear particularly promising; however, the current evidence remains preliminary due to the limited number of available studies. High-quality evidence is especially scarce for smartphone and gaming addictions. Future research should prioritize methodological rigor, expand intervention trials for underrepresented addiction subtypes, and develop tailored strategies based on distinct addiction profiles. Collectively, these findings provide an evidence-based foundation for designing more precise, scalable, and context-sensitive interventions in both clinical and educational settings.

## Supplementary material

10.2196/81705 Multimedia Appendix 1 Search strategy; Excluded studies; characteristics of the included studies; A Measurement Tool to Assess Systematic Reviews 2; and Grading of Recommendations Assessment, Development, and Evaluation tool.

10.2196/81705 Checklist 1 PRISMA checklist.

## Abbreviations

AMSTAR 2: A Measurement Tool to Assess Systematic Reviews 2
CBT: cognitive behavioral therapy
GRADE: Grading of Recommendations Assessment, Development, and Evaluation
*ICD-11*: *International Classification of Diseases, 11th Revision*
PRISMA: Preferred Reporting Items for Systematic Reviews and Meta-Analyses
RCT: randomized controlled trial
SMD: standardized mean difference
