# Supplementary material for: Effectiveness of Interventions for Internet, Smartphone, and Gaming Addictions: Umbrella Review and Meta–Meta-Analysis
Source: J Med Internet Res. 2026 Mar 5;28:e81705. doi: 10.2196/81705 (PMC12978896; doi:10.2196/81705)
Supplement: Multimedia Appendix 1 [file jmir-v28-e81705-s001.doc]

**Supplementary S1. The search strategy**

**Pubmed**

| #1 | Search: ((((((((((((((((Internet Addiction Disorders[MeSH Terms]) OR (Internet Addiction[Title/Abstract])) OR (Social Media Addiction[Title/Abstract])) OR (Smartphone Addiction[Title/Abstract])) OR (Internet Gaming Disorder[Title/Abstract])) OR (Addictive internet use[Title/Abstract])) OR (Gaming disorder[Title/Abstract])) OR (Excessive internet use[Title/Abstract])) OR (online addiction[Title/Abstract])) OR (Pathological Internet[Title/Abstract])) OR (Problematic internet use[Title/Abstract])) OR (Problematic smartphone use[Title/Abstract])) OR (Problematic social media[Title/Abstract])) OR (Compulsive Internet Use[Title/Abstract])) OR (Internet Use Disorder[Title/Abstract]))) |
| --- | --- |
| #2 | Search: (((Intervention[MeSH Terms]) OR (Treatment[MeSH Terms])) OR (Therapy[MeSH Terms]))) |
| #3 | Search: ( (meta-analysis[Publication Type]) OR(meta-[Publication Type])) |
| #4 | #1 AND #2 AND #3 |

**Wed of Science**

((((((((((((((TS=(Internet Addiction Disorders)) OR TI=(Internet Addiction)) OR TI=(Social Media Addiction)) OR TI=(Smartphone Addiction)) OR TI=(Internet Gaming Disorder)) OR TI=(Addictive internet use)) OR TI=(Gaming disorder)) OR TI=(Excessive internet use)) OR TI=(online addiction)) OR TI=(Pathological Internet)) OR TI=(Problematic internet use)) OR TI=(Problematic smartphone use)) OR TI=(Problematic social media)) OR TI=(Compulsive Internet Use)) OR TI=(Internet Use Disorder)

AND

(((TS=(Intervention)) OR TI=(Treatment)) OR TI=(Therapy)) OR TI=(therapeutic)

AND

(TS=(Meta-analysis)) OR TS=(meta-)

**Cochrane Library**

| #1 | MeSH descriptor: [Internet Addiction Disorder] explode all trees |
| --- | --- |
| #2 | Internet Addiction |
| #3 | Social Media Addiction |
| #4 | Smartphone Addiction |
| #5 | Internet Gaming Disorder |
| #6 | Addictive internet us |
| #7 | Gaming disorder |
| #8 | Excessive internet use |
| #9 | Pathological Internet |
| #10 | Problematic internet use |
| #11 | Problematic smartphone use |
| #12 | Problematic social media |
| #13 | Compulsive Internet Use |
| #14 | Internet Use Disorder |
| #15 | #1 OR #2 OR #3 OR #4 OR #5 OR #6 OR #7 OR #8 OR #9 OR #10 OR #11 OR #12 OR #13 OR #14 |
| #16 | MeSH descriptor: [] explode all trees |
| #17 | intervention |
| #18 | treatment |
| #19 | therapy |
| #20 | #16 OR #17 OR #18 OR #19 |
| #21 | #15 AND #20 |

**Scopus**

TITLE-ABS-KEY(Internet Addiction Disorders) OR TITLE-ABS-KEY(Internet Addiction) OR TITLE-ABS-KEY(Social Media Addiction) OR TITLE-ABS-KEY(Smartphone Addiction) OR TITLE-ABS-KEY(Internet Gaming Disorder) OR TITLE-ABS-KEY(Addictive internet use) OR TITLE-ABS-KEY(Gaming disorder) OR TITLE-ABS-KEY(Excessive internet use) OR TITLE-ABS-KEY(online addiction) OR TITLE-ABS-KEY(Pathological Internet) OR TITLE-ABS-KEY(Problematic internet use) OR TITLE-ABS-KEY(Problematic smartphone use) OR TITLE-ABS-KEY(Problematic social media) OR TITLE-ABS-KEY(Compulsive Internet Use) OR TITLE-ABS-KEY(Internet Use Disorder) AND TITLE-ABS-KEY(Intervention) OR TITLE-ABS-KEY(Treatment) OR TITLE-ABS-KEY(Therapy) AND TITLE-ABS-KEY(meta-analysis)

**APA**
subject(Internet Addiction Disorders) OR subject(Internet Addiction) OR subject(Smartphone Addiction) OR subject(Internet Gaming Disorder) OR subject(Addictive internet use) OR subject(Gaming disorder) OR subject(Excessive internet use) OR subject(Pathological Internet) OR subject(Problematic internet use) OR subject(Problematic smartphone use) AND [subject(Intervention) OR subject(Treatment) OR subject(Therapy)](https://www.proquest.com/recentsearches.recentsearchtabview.recentsearchesgridview.scrolledrecentsearchlist.checkdbssearchlink:rerunsearch/ECE16276243C49C5PQ/None/$N?_csrf=8bc7e2c5-3e69-454a-bdde-86f20e3b3ce8&site=psycarticles&t:ac=RecentSearches) AND [subject(meta-analysis)](https://www.proquest.com/recentsearches.recentsearchtabview.recentsearchesgridview.scrolledrecentsearchlist.checkdbssearchlink:rerunsearch/570C21331F154872PQ/None/$N?_csrf=8bc7e2c5-3e69-454a-bdde-86f20e3b3ce8&site=psycarticles&t:ac=RecentSearches)

**Supplementary Table S2. Excluded studies.**

| Exclude Reason | Study |
| --- | --- |
| Overall review | Basenach L, Renneberg B, Salbach H, Dreier M, Woelfling K. Systematic reviews and meta-analyses of treatment interventions for Internet use disorders: Critical analysis of the methodical quality according to the PRISMA guidelines. Journal of behavioral addictions. 2023;12(1):9-25. |
| Drug control | Chang C-H, Chang Y-C, Yang L, Tzang R-F. The Comparative Efficacy of Treatments for Children and Young Adults with Internet Addiction/Internet Gaming Disorder: An Updated Meta-Analysis. International journal of environmental research and public health. 2022;19(5). |
| No English | Choi D, Youngkeun K. Meta-Analysis on the Effect of Group Consultation Program on Smartphone Addiction Treatment for University Students |
| No English | Gwi Im J, Hoi KJ. Meta Analysis on the of Internet and Smart-phone Addiction Group Counseling Program |
| No English | Jung KE, Jang S-O. A meta-analysis on the effects of promoting self-control group program for internet addiction youth |
| No intervention | Ma Y, Zhou Z, Ye C, Liu M. Online social support and problematic Internet Use-a meta-analysis. ADDICTIVE BEHAVIORS. 2025;160. |
| No intervention | Meynadier J, Malouff JMM, Loi NMM, Schutte NSS. Lower Mindfulness is Associated with Problematic Social Media Use: A Meta-Analysis. CURRENT PSYCHOLOGY. 2024;43(4):3395-404. |
| No English | Ock C-M, Lee H-S, Kim H. Effectiveness of CBT-based interventions for internet gaming disorder (IGD): A systematic review and meta-analysis |
| No English | Ock C-M, Lee H-S, Kim H. Effectiveness of School-based Prevention Programs on Internet Gaming Disorder Severity in Adolescents: A Systematic Review and Meta-analysis |
| No English | oh I, Kim C. Meta-analysis on the effects of the prevention and intervention programs for internet addiction |
| No meta-analysis | Pirwani N, Szabo A. Could physical activity alleviate smartphone addiction in university students? A systematic literature review. PREVENTIVE MEDICINE REPORTS. 2024;42. |
| No intervention | Ru Y, Norlizah HC, Burhanuddin NAN, Liu H, Dong J. The correlation between mindfulness and problematic smartphone use: A meta-analysis. ADDICTIVE BEHAVIORS. 2025;164. |
| No intervention | Saletti SMR, Van den Broucke S, Chau C. The Effectiveness of Prevention Programs for Problematic Internet Use in Adolescents and Youths: A Systematic Review and Meta-Analysis. CYBERPSYCHOLOGY-JOURNAL OF PSYCHOSOCIAL RESEARCH ON CYBERSPACE. 2021;15(2). |
| No meta-analysis | Sharma M, Palanichamy T. Psychosocial interventions for technological addictions. Indian Journal of Psychiatry, suppl Supplement 4. 2018;60(8). |
| No meta-analysis | Soon KM, Han S-J, Yeun Y-R. Effectiveness of Smartphone Addiction Intervention for University Students: A Systematic Review and Meta Analysis. Journal of the Korea Convergence Society. 2020;11(5):399-412. |
| No meta-analysis | Theopilus Y, Al Mahmud A, Davis H, Octavia JR. Preventive Interventions for Internet Addiction in Young Children:Systematic Review. JMIR MENTAL HEALTH. 2024;11. |
| No intervention | Throuvala MA, Griffiths MD, Rennoldson M, Kuss DJ. School-based prevention for adolescent internet addiction: Prevention is the key. A systematic literature review. Current Neuropharmacology. 2019;17(6):507-25. |
| No English | Yang J, 김현실. A meta-analysis of effects of smartphone addiction prevention program for adolescents |
| No English | Yeon HJ, Sung-Man S. The Effectiveness of Youth Internet Addiction Group Counseling Programs: a Meta-Analysis |
| No intervention | Zhang J, Zhang Q, Xiao B, Cao Y, Chen Y, Li Y. Parental technoference and child problematic media use: Meta-analysis. Journal of medical Internet research. 2025;27:16. |

**Supplementary Table S3. Characteristics of included studies.**

| **Author** | **Year** | **Country** | **Sample** | **Female (%)** | **Age** | **Study N** | **Addiction type** | **Interventions** | **Controls** | **Design** |
| --- | --- | --- | --- | --- | --- | --- | --- | --- | --- | --- |
| Augner(1) | 2022 | Austria | 1439 | NA | 20.34±6.63 | 10 | Problematic Internet use Problematic Smartphone use | Psychological | Mixed | Mixed |
| Chun(2) | 2017 | South Korea | NA | NA | adolescents | 70 | Internet Addiction | Psychological | Mixed | Mixed |
| Danielsen(3) | 2024 | Norway | 9524 | NA | 9.87-27 | 38 | Gaming disorder | Psychotherapy Behavioral | Mixed | Mixed |
| Gao(4) | 2025 | China | 1282 | NA | 10-20 | 20 | Internet Addiction | Exercise | No intervention | RCT |
| Goslarp(5) | 2020 | Austria | 2427 | NA | 21 | 91 | Internet Addiction | Psychological | Mixed | RCT |
| Jiang(6) | 2024 | China | 4385 | NA | youth | 66 | Internet Addiction | non-pharmacological | Mixed | RCT |
| Jing(7) | 2025 | China | 5986 | NA | 12-26 | 90 | Problematic Internet use | Exercise Psychological Bio feedback | No intervention | RCT |
| Kim(8) | 2022 | South Korea | 745 | NA | 11-52 | 17 | Excessive gaming | Psychological | Mixed | Mixed |
| Kim(9) | 2019 | South Korea | 658 | NA | 11-56 | 11 | Internet Addiction | Psychological | Mixed | Mixed |
| Kurnaz(10) | 2024 | Turkey | 694 | NA | NA | 15 | Internet Addiction Gaming addiction Internet use disorder | Psychological | Mixed | Mixed |
| Liu(11) | 2017 | China | 2871 | NA | NA | 58 | Internet Addiction | Psychological | Mixed | RCT |
| Liu(12) | 2019 | China | 1582 | NA | 20.43 | 9 | Smartphone Addiction | Exercise | Mixed | RCT |
| Lo(13) | 2023 | China | 2218 | NA | NA | 34 | Internet-related Addiction | Psychosocial | No intervention Active control | Mixed |
| Malinauskas(14) | 2019 | Lithuania | 305 | NA | adolescents | 6 | Internet Addiction | Psychological | No intervention | RCT |
| Ock(15) | 2025 | South Korea | 1950 | NA | 10-30 | 19 | Gaming disorder | Non-pharmacological | Mixed | RCT |
| Reangsing(16) | 2025 | USA | 1115 | NA | 19.05 | 13 | Gaming disorder | Psychological | Mixed | Mixed |
| Soon(17) | 2020 | South Korea | 915 | NA | NA | 13 | Smartphone Addiction | Psychological | Mixed | Mixed |
| Stevens(18) | 2018 | Australia | 580 | NA | NA | 12 | Gaming disorder | Psychological | Mixed | Mixed |
| Vicente(19) | 2025 | Spain | 782 | 59.58 | 24.31 | 8 | Internet Addiction | Psychological | Mixed | Mixed |
| Wang(20) | 2024 | China | 1587 | NA | NA | 18 | Internet Addiction | Psychological | Mixed | Mixed |
| Wang(21) | 2023 | China | 1738 | NA | NA | 32 | Gaming disorder | Psychological | Mixed | Mixed |
| Yan(22) | 2025 | China | 760 | NA | college students | 14 | Internet Addiction | Psychological | Mixed | RCT |
| Yuen(23) | 2016 | South Korea | 1490 | NA | children | 37 | Internet Addiction | Psychological | Mixed | Mixed |
| Zhang(24) | 2023 | China | 2367 | NA | NA | 26 | Internet Addiction | Psychological Exercise | Mixed | Mixed |
| Zhang(25) | 2024 | China | 2476 |  | college students | 38 | Internet Addiction | Psychological Exercise | Mixed | Mixed |
| Zhang(26) | 2022 | China | 3832 | NA | NA | 59 | Internet Addiction | Psychological Exercise | No intervention | RCT |
| Zhang(27) | 2023 | China | 2408 | NA | NA | 39 | Internet Addiction | Exercise | No intervention | RCT |
| Zhou(28) | 2024 | China | 6876 |  | college students | 89 | Internet Addiction | Exercise | Mixed | Mixed |
| Zhu(29) | 2023 | China | 3538 | NA | NA | 57 | Internet Addiction | Behavioral | No intervention | RCT |

Note: NA: not available.

# **Supplementary Table S4.** AMSTAR-2 risk of bias.

| **Author** | **Year** | **1** | **2** | **3** | **4** | **5** | **6** | **7** | **8** | **9** | **10** | **11** | **12** | **13** | **14** | **15** | **16** | **Rating** |
| --- | --- | --- | --- | --- | --- | --- | --- | --- | --- | --- | --- | --- | --- | --- | --- | --- | --- | --- |
| Augner | 2022 | Yes | No | No | Yes | Yes | Yes | No | Yes | No | No | No | No | No | Yes | Yes | Yes | Critically low |
| Chun | 2017 | Yes | No | No | Partial Yes | No | No | No | No | No | No | No | No | No | Yes | Yes | Yes | Critically low |
| Danielsen | 2024 | Yes | Yes | No | Yes | Yes | Yes | Yes | Yes | Yes | Yes | No | Yes | Yes | Yes | Yes | Yes | Low |
| Gao | 2025 | Yes | No | Yes | Yes | Yes | Yes | No | Yes | Partial Yes | No | Yes | Yes | Yes | Yes | Yes | Yes | Critically low |
| Goslarp | 2020 | Yes | No | No | Yes | Yes | Yes | No | Yes | Yes | No | Yes | No | No | Yes | Yes | Yes | Critically low |
| Jiang | 2024 | Yes | No | Yes | Partial Yes | Yes | No | Yes | Yes | Yes | No | Yes | No | No | Yes | Yes | Yes | Critically low |
| Jing | 2025 | Yes | No | Yes | Partial Yes | Yes | No | Yes | Yes | Yes | No | Yes | No | No | Yes | Yes | Yes | Critically low |
| Kim | 2022 | No | No | No | Yes | Yes | Yes | No | Yes | Yes | No | Yes | Yes | Yes | Yes | Yes | Yes | Critically low |
| kim | 2019 | No | No | No | Partial Yes | No | Yes | No | No | No | No | Yes | Yes | Yes | Yes | Yes | Yes | Critically low |
| Kurnaz | 2024 | Yes | Yes | Yes | Partial Yes | No | No | No | Yes | No | No | Yes | Yes | Yes | Yes | Yes | Yes | Critically low |
| Liu | 2017 | Yes | No | No | Yes | Yes | Yes | No | Yes | Yes | No | Yes | No | Yes | Yes | Yes | Yes | Critically low |
| Liu | 2019 | Yes | No | No | Partial Yes | Yes | Yes | No | Yes | Yes | No | Yes | No | Yes | Yes | Yes | Yes | Critically low |
| Lo | 2023 | Yes | No | Yes | Yes | Yes | No | No | Yes | Partial Yes | Yes | Yes | Yes | Yes | Yes | Yes | Yes | Critically low |
| Malinauskas | 2019 | Yes | No | No | Yes | Yes | Yes | No | Yes | Partial Yes | No | Yes | No | No | Yes | Yes | Yes | Critically low |
| Ock | 2025 | Yes | No | No | Partial Yes | No | Yes | No | Yes | Partial Yes | No | Yes | No | Yes | Yes | Yes | Yes | Critically low |
| Reangsing | 2025 | Yes | No | No | Partial Yes | Yes | Yes | No | Yes | No | Yes | Yes | No | Yes | Yes | Yes | Yes | Critically low |
| Soon | 2020 | Yes | No | No | Partial Yes | No | Yes | No | Yes | No | No | No | No | Yes | Yes | Yes | Yes | Critically low |
| Stevens | 2018 | Yes | No | Yes | Yes | Yes | Yes | No | Yes | Partial Yes | No | Yes | No | No | Yes | Yes | Yes | Critically low |
| Vicente | 2025 | Yes | Yes | Yes | Yes | No | Yes | No | Yes | No | No | Yes | Yes | Yes | Yes | Yes | Yes | Critically low |
| Wang | 2024 | Yes | Yes | Yes | Yes | No | Yes | No | Yes | Yes | Yes | No | Yes | Yes | Yes | Yes | Yes | Critically low |
| Wang | 2023 | Yes | Yes | Yes | Yes | Yes | Yes | No | Yes | Partial Yes | Yes | No | Yes | Yes | Yes | Yes | Yes | Critically low |
| Yan | 2025 | Yes | No | Yes | Yes | Yes | No | No | Yes | Partial Yes | Yes | Yes | Yes | Yes | Yes | Yes | Yes | Critically low |
| Yuen | 2016 | Yes | No | No | Yes | Yes | No | No | Yes | Partial Yes | No | No | No | No | Yes | No | Yes | Critically low |
| Zhang | 2023 | Yes | Yes | No | Yes | Yes | Yes | No | Yes | Yes | Yes | Yes | Yes | Yes | Yes | Yes | Yes | Low |
| Zhang | 2024 | Yes | Yes | No | Yes | Yes | Yes | No | Yes | Yes | Yes | Yes | Yes | Yes | Yes | Yes | Yes | Low |
| Zhang | 2022 | Yes | Yes | No | Yes | Yes | Yes | No | Yes | Yes | No | Yes | Yes | Yes | Yes | Yes | Yes | Low |
| Zhang | 2023 | Yes | Yes | No | Yes | Yes | Yes | No | Yes | Yes | Yes | Yes | Yes | No | No | Yes | Yes | Critically low |
| Zhou | 2024 | Yes | Yes | Yes | Yes | Yes | Yes | No | Yes | Partial Yes | Yes | No | Yes | Yes | Yes | Yes | Yes | Critically low |
| Zhu | 2023 | Yes | No | Yes | Yes | Yes | No | No | Yes | Partial Yes | Yes | Yes | Yes | Yes | Yes | Yes | Yes | Critically low |

AMSTAR-2 Items

1. Did the research questions and inclusion criteria for the review include the components of PICO?

2. Did the report of the review contain an explicit statement that the review methods were established prior to the conduct of the review and did the report justify any significant deviations from the protocol?

3. Did the review authors explain their selection of the study designs for inclusion in the review?

4. Did the review authors use a comprehensive literature search strategy?

5. Did the review authors perform study selection in duplicate?

6. Did the review authors perform data extraction in duplicate?

7. Did the review authors provide a list of excluded studies and justify the exclusions?

8. Did the review authors describe the included studies in adequate detail?

9. Did the review authors use a satisfactory technique for assessing the risk of bias (RoB) in individual studies that were included in the review?

10. Did the review authors report on the sources of funding for the studies included in the review?

11. If meta-analysis was performed did the review authors use appropriate methods for statistical combination of results?

12. If meta-analysis was performed, did the review authors assess the potential impact of RoB in individual studies on the results of the meta-analysis or other evidence synthesis?

13. Did the review authors account for RoB in individual studies when interpreting/ discussing the results of the review?

14. Did the review authors provide a satisfactory explanation for, and discussion of, any heterogeneity observed in the results of the review?

15. If they performed quantitative synthesis did the review authors carry out an adequate investigation of publication bias (small study bias) and discuss its likely impact on the results of the review?

16. Did the review authors report any potential sources of conflict of interest, including any funding they received for conducting the review?

# **Supplementary Table S5. GRADE summary table.**

| **Author** | **Year** | **Outcome** | **Factors that may decrease and increase certainty of evidence** | | | | | **GRADE** |
| --- | --- | --- | --- | --- | --- | --- | --- | --- |
|  |  |  | **Risk of bias** | **Indirectness** | **Inconsistency** | **Imprecision** | **Publication bias** |  |
| Augner | 2022 | Problematic Internet use Problematic Smartphone use | Y | Y | N | Y | Y | ⬤◯ ◯ ◯ Very Low |
| Chun | 2017 | Internet Addiction | Y | Y | N | Y | Y | ⬤◯ ◯ ◯ Very Low |
| Danielsen | 2024 | Gaming disorder | Y | Y | N | N | Y | ⬤◯ ◯ ◯ Very Low |
| Gao | 2025 | Internet Addiction | N | Y | N | N | Y | ⬤⬤ ◯ ◯ Low |
| Goslarp | 2020 | Internet Addiction | N | Y | N | N | Y | ⬤⬤ ◯ ◯ Low |
| Jiang | 2024 | Internet Addiction | N | Y | N | Y | Y | ⬤◯ ◯ ◯ Very Low |
| Jing | 2025 | Problematic Internet use | N | Y | N | Y | Y | ⬤◯ ◯ ◯ Very Low |
| Kim | 2022 | Excessive gaming | Y | Y | N | Y | Y | ⬤◯ ◯ ◯ Very Low |
| Kim | 2019 | Internet Addiction | Y | Y | N | N | Y | ⬤◯ ◯ ◯ Very Low |
| Kurnaz | 2024 | Internet Addiction Gaming addiction Internet use disorder | Y | Y | N | Y | Y | ⬤◯ ◯ ◯ Very Low |
| Liu | 2017 | Internet Addiction | N | Y | N | Y | Y | ⬤◯ ◯ ◯ Very Low |
| Liu | 2019 | Smartphone Addiction | N | Y | N | Y | Y | ⬤◯ ◯ ◯ Very Low |
| Lo | 2023 | Internet-related Addiction | Y | N | N | Y | Y | ⬤◯ ◯ ◯ Very Low |
| Malinauskas | 2019 | Internet Addiction | N | Y | N | Y | Y | ⬤◯ ◯ ◯ Very Low |
| Ock | 2025 | Gaming disorder | N | N | N | N | Y | ⬤⬤⬤ ◯ Moderate |
| Reangsing | 2025 | Gaming disorder | Y | Y | N | Y | Y | ⬤◯ ◯ ◯ Very Low |
| Soon | 2020 | Smartphone Addiction | Y | Y | N | Y | Y | ⬤◯ ◯ ◯ Very Low |
| Stevens | 2018 | Gaming disorder | Y | Y | N | N | Y | ⬤◯ ◯ ◯ Very Low |
| Vicente | 2025 | Internet Addiction | Y | Y | N | Y | Y | ⬤◯ ◯ ◯ Very Low |
| Wang | 2024 | Internet Addiction | Y | Y | N | N | Y | ⬤◯ ◯ ◯ Very Low |
| Wang | 2023 | Gaming disorder | Y | Y | N | N | Y | ⬤◯ ◯ ◯ Very Low |
| Yan | 2025 | Internet Addiction | N | Y | N | N | Y | ⬤⬤ ◯ ◯ Low |
| Yuen | 2016 | Internet Addiction | Y | Y | N | Y | Y | ⬤◯ ◯ ◯ Very Low |
| Zhang | 2023 | Internet Addiction | Y | Y | N | Y | Y | ⬤◯ ◯ ◯ Very Low |
| Zhang | 2024 | Internet Addiction | Y | Y | N | Y | Y | ⬤◯ ◯ ◯ Very Low |
| Zhang | 2022 | Internet Addiction | N | Y | N | N | Y | ⬤⬤ ◯ ◯ Low |
| Zhang | 2023 | Internet Addiction | N | Y | N | N | Y | ⬤⬤ ◯ ◯ Low |
| Zhou | 2024 | Internet Addiction | Y | Y | Y | Y | Y | ⬤◯ ◯ ◯ Very Low |
| Zhu | 2023 | Internet Addiction | N | Y | N | N | Y | ⬤⬤ ◯ ◯ Low |

**Figure S1**. Funnel plot with filled points.

**Figure S2**. Forest plot for overall interventions.

**Figure S3**. Forest plot for overall controls.

**Figure S4**. Forest plot for overall study design.

**Figure S5**. Forest plot of Internet addiction for interventions.

**Figure S6**. Forest plot of Internet addiction for controls.

**Figure S7**. Forest plot of Internet addiction for study design.

**Figure S8**. Forest plot of Internet gaming addiction for intervention

**Figure S9**. Forest plot of Internet gaming addiction for study design.

**Figure S10**. Forest plot of smartphone addiction for interventions

**Figure S11**. Forest plot of smartphone addiction for study design

**References**

1. Augner C, Vlasak T, Aichhorn W, Barth A. Tackling the 'digital pandemic': The effectiveness of psychological intervention strategies in problematic Internet and smartphone use-A meta-analysis. AUSTRALIAN AND NEW ZEALAND JOURNAL OF PSYCHIATRY. 2022;56(3):219-29.

2. Chun J, Shim H, Kim S. A meta-analysis of treatment interventions for internet addiction among Korean adolescents. Cyberpsychology, Behavior, and Social Networking. 2017;20(4):225-31.

3. Danielsen PA, Mentzoni RA, Låg T. Treatment effects of therapeutic interventions for gaming disorder: A systematic review and meta-analysis. Addictive Behaviors. 2024;149:1-17.

4. Gao W, Zhou H, Zou X, Li M, Lin X. The impact of exercise and exercise-based combined therapies on adolescent internet addiction: A systematic review and network meta-analysis. Current Psychology: A Journal for Diverse Perspectives on Diverse Psychological Issues. 2025.

5. Goslarp M, Leibetseder M, Muench HM, Hofmann SG, Laireiter A-R. Treatments for internet addiction, sex addiction and compulsive buying: A meta-analysis. Journal of behavioral addictions. 2020;9(1):14-43.

6. Jiang Y-S, Liu T-H, Qin D, Wang Z-P, He X-Y, Chen Y-N. Effects of non-pharmacological interventions on youth with internet addiction: a systematic review and meta-analysis of randomized controlled trials. Frontiers in psychiatry. 2024;14.

7. Jing-Jing T, Xiao-Ya H, Guo Z. Optimal Non-Pharmacological Interventions for Reducing Problematic Internet Use in Youth: A Systematic Review and Bayesian Network Meta-Analysis. Behavioral Sciences. 2025;15(1):98.

8. Kim J, Lee S, Lee D, Shim S, Balva D, Choi K-H, et al. Psychological treatments for excessive gaming: a systematic review and meta-analysis. Scientific Reports (Nature Publisher Group). 2022;12(1).

9. Kim S, Noh D. The Current Status of Psychological Intervention Research for Internet Addiction and Internet Gaming Disorder. Issues in Mental Health Nursing. 2019;40(4):335-41.

10. Kurnaz MF, Kocturk N. Are CBT-based interventions effective for pathologic technology use? A meta-analysis of experimental studies. CURRENT PSYCHOLOGY. 2024;43(12):11127-38.

11. Liu J, Nie J, Wang Y. Effects of Group Counseling Programs, Cognitive Behavioral Therapy, and Sports Intervention on Internet Addiction in East Asia: A Systematic Review and Meta-Analysis. International journal of environmental research and public health. 2017;14(12):1470.

12. Liu S, Xiao T, Yang L, Loprinzi PD. Exercise as an Alternative Approach for Treating Smartphone Addiction: A Systematic Review and Meta-Analysis of Random Controlled Trials. International journal of environmental research and public health. 2019;16(20).

13. Lo CKM, Chan KL, Yu L, Chui WWH, Ip P. Long-term effects of psychosocial interventions on internet-related disorders: A meta-analysis. COMPUTERS IN HUMAN BEHAVIOR. 2023;138.

14. Malinauskas R, Malinauskiene V. A meta-analysis of psychological interventions for Internet/smartphone addiction among adolescents. Journal of behavioral addictions. 2019;8(4):613-24.

15. Ock C-M, Lee H-S, Chae J, Kim H. Effectiveness of Non-Pharmacological Interventions on Gaming Disorder: A Systematic Review and Meta-Analysis. PSYCHIATRY INVESTIGATION. 2025;22(5):490-503.

16. Reangsing C, Wongchan W, Trakooltorwong P, Thaibandit J, Oerther S. Effects of cognitive behavioral therapy (CBT) on addictive symptoms in individuals with internet gaming disorders: A systematic review and meta-analysis. Psychiatry research. 2025;348.

17. Soon KM, Han S-J, Yeun Y-R. Effectiveness of Smartphone Addiction Intervention for University Students: A Systematic Review and Meta Analysis. Journal of the Korea Convergence Society. 2020;11(5):399-412.

18. Stevens MWR, King DL, Dorstyn D, Delfabbro PH. Cognitive-behavioral therapy for Internet gaming disorder: A systematic review and meta-analysis. CLINICAL PSYCHOLOGY & PSYCHOTHERAPY. 2019;26(2):191-203.

19. Vicente-Escudero JL. Paradox or efficacy. Meta-analysis of internet-based psychological interventions to reduce internet addiction, anxiety and depression in internet-addicted people. CURRENT PSYCHOLOGY. 2025.

20. Wang H, Li X, Lok GKI, Meng C, Tan Y, Lee UM, et al. Family-based therapy for internet addiction among adolescents and young adults: A meta-analysis. Journal of behavioral addictions. 2024;13(2).

21. Wang X, Zhang Y, Lin J, Wong ACW, Chan KK-y, Wong SY-s, et al. Treatments of internet gaming disorder and comorbid mental disorders: A systematic review and meta-analysis. Computers in Human Behavior. 2023;149:1-9.

22. Yan Y, Qin X, Liu L, Zhang W, Li B. Effects of exercise interventions on Internet addiction among college students: A systematic review and meta-analysis of randomized controlled trials. Addict Behav. 2025;160:108159.

23. Yeun Y-R, Suk-Jung H. Effects of Psychosocial Interventions for Schoolaged Children’s Internet Addiction, Self-control and Self-esteem: Meta-Analysis. Healthcare Informatics Research. 2016;22(3):217-30.

24. Zhang K, Lu X, Zhang X, Zhang J, Ren J, Guo H, et al. Effects of Psychological or Exercise Interventions on Problematic Mobile Phone Use: a Systematic Review and Meta-analysis. Current Addiction Reports. 2023;10(2):230-53.

25. Zhang M, Meng S-Q, Hasan AJ, Han Y, Han S, Li B, et al. Network meta-analysis of the effectiveness of different interventions for internet addiction in college students. Journal of affective disorders. 2024;363:26.

26. Zhang X, Zhang J, Zhang K, Ren J, Lu X, Wang T, et al. Effects of different interventions on internet addiction: A meta-analysis of random controlled trials. Journal of affective disorders. 2022;313:56-71.

27. Zhang Y, Li G, Liu C, Chen H, Guo J, Shi Z. Mixed comparison of interventions for different exercise types on students with Internet addiction: a network meta-analysis. Frontiers in psychology. 2023;14.

28. Zhou Z, Wan Y, Li C, Yuan J, Gao G, Cui H, et al. Effectiveness of sports intervention: A meta-analysis of the effects of different interventions on adolescent internet addiction. Journal of affective disorders. 2024;365:644.

29. Zhu Y, Chen H, Li J, Xian M, Wang W. Effects of different interventions on internet addiction: a systematic review and network meta-analysis. BMC psychiatry. 2023;23:1-17.
